# Progress in Medicine

**Published:** 1897-02-13

**Authors:** 


### Progress in Medicine.

### KIDNEY DISEASES.

**Kidney Tension.**—The suggestion of surgical interference in some forms of Bright's disease made by Reginald Harrison nearly a year ago is based on the relief of that tension which occurs in acute inflammation, and which otherwise may cause atrophy and degeneration of the secreting structures. In a fresh paper, Mr. Harrison[1] refers to the effects of inflammation in the eye and testicle, two organs which are likewise enclosed by an unyielding capsule, and brings forward some new cases, showing the distended condition of certain inflamed kidneys, the rapidly increased excretion of urine which followed puncture, and the reflex improvement in the second kidney after puncture of one. He thinks the operation may be useful where severe attacks (as sometimes seen in scarlatina) produce suppression, or where, later on, no improvement takes place, and the kidney is tender under pressure. David Newman[2] points out that increased tension in the kidney may be due—(*a*) To torsion of the renal vessels and the ureter, with pain, hæmaturia, and albuminuria; (*b*) irritation either direct or reflex, as from a strangulated hernia. He is disposed to regard kidney hyperæmia as of frequent occurrence in acute intestinal obstruction. E. Hurry Fenwick[3] objects to digital exploration, as the renal tissue is friable beyond belief in acute states, and breaks to pieces at the slightest touch with often serious hæmorrhage, while a kidney which has quieted down may relapse into an acute state. Still, he finds that puncture and section of part of the capsule are of much value. Bronowski[4] reports that strontium lactate proves itself an invaluable diuretic, especially in acute nephritis. The quantity of water is always increased, and in acute cases the albumen is diminished, but there is no change in the pulse or in the quantity of the ethereal sulphates. Lemoine[5] recommends an extract of the inner bark of elder (sambucus niger) as a diuretic, which has no congestive action on the kidney. Both in kidney and heart disease it has given good results, and may be administered with digitalis. Hæmaturia from healthy kidneys was not improbably the condition in some of Harrison's cases. Klemperer,[6] at Berlin, brought forward some undisputed instances, and considers that such hæmorrhage may occur from over-exertion or fatigue, just as epistaxis does, or from hæmophilia, or occasionally from neurotic causes. Other speakers confirmed the fact of its occurrence with perfectly healthy kidneys, and its cessation after surgical intervention for supposed calculus, or when rest was enforced.

**Urine Analysis.**—Kast and Weiss[7] deny the conclusions of Stokvis that hæmato-porphyrinuria is readily produced in rabbits by sulphonal as a result of extravasations in the mucous membrane of the stomach. They repeated his experiments, and found that when a dark colouring matter appeared in rabbits' urine it was not of this nature. Still, hæmato-porphyrin may occur after sulphonal has been given to human beings in a state of anæmia and constipation, though very rarely. Simple fibrinuria has only been recorded in five cases, one of which is discussed by A. Klein.[8] The patient suffered from Bright's disease, and occasionally large masses of fibrin appeared in the water, which clotted when shaken like serous fluid. The urine at this time was alkaline, contained much albumen, no blood or chyle, and hardly any phosphates. The patient had suffered previously from rigors, and after death the kidneys were found to have undergone amyloid degeneration. The importance of bacteria in the urine is lessened by the researches of Chvostek and Egger,[9] who find they are excreted in large quantities in non-bacterial febrile states, such as malarial paroxysms. The fever appears to act physiologically in thus expelling from the body any bacteria which have accidentally gained entrance. Another curious excretion of anthracite coal dust was also observed by Betz in the urine of a stove cleaner. Vaughan Harley,[10] in discussing the origin of urobilin, favours the view that it is formed from the bilirubin in the intestines, and absorbed thence. This process chiefly occurs when intestinal putrefaction is active, and when blood destruction or intestinal hæmorrhage has taken place. A case of chyluria is reported by H. J. Daggett,[11] where the patient passed a milky fluid, alkaline, and with coagula, for three years without any impairment of health. He had never been out of England and the cause was unknown. Physiological albuminuria was absent in every one of one hundred and forty-four healthy individuals examined by Zeehuisek.[12] Five per cent. of such cases of albuminuria are due, he thinks, to some renal mischief, and others to extra renal causes. Arnozan,[13] while allowing that all albuminuria is pathological, regards it as only of relative importance. It may arise from an alteration of the blood, a change in the blood tension in the kidney, a lesion in the glomerular epithelium, or an inflammation of the convoluted tubules. Indeed, all epithelia of the body exude albumen when inflamed, and in albuminuria we have no indication where the lesion is, or as to its nature. Thus the formed blood constituents found in the urine and its toxicity are of greater importance than the amount of albumen. The use of many modern drugs interferes with the reaction of certain ordinary tests. Thus, Esbach and Tanret's tests for albumen are interfered with by antipyrine, the presence of which can be detected by the green colour that appears on the addition of strong nitric acid. The tests for some forty of these drugs in the urine are given in an admirable paper by J. R. Moechel,[14] which is a storehouse of information. Leidlie[15] points out that pyine and mucin do not exist as such in urine containing pus before ammoniacal fermentation. Pyine is an alkali-albumen and mucin a nucleo-albumen. Elliott[16] recommends as a routine test for serum albumen, which will not react to any other albuminous body such as albumose, the common ferro-cyanide solution. He adds a drachm of it to two drachms of urine, and not till afterwards a little acetic acid, and finds it perfectly reliable. For detection of other proteids he advises the following procedure: Add a few drops of acetic acid with a drachm of potassio-mercuric-iodide to half a test tube of urine. If no precipitate occurs, there is no form or any trace of albumen present. Otherwise, heat, and if the precipitate is not dissolved we have either serum albumen (for which the ferro-cyanide test suffices), an oleo-resin, the precipitate of which is dissolved by alcohol, or mucin, which is not so dissolved. If the original cloud did disappear on heating we have either peptones, shown by the sulphate ammonium test; proteose, which is unaffected by it; or alkaloids, where alone the cloud is dissolved by ether. The special peptone test is carried out by saturating slightly acidulated urine with sulphate of ammonium, filtering off any precipitate, and adding potassio-mercuric-iodide, when peptone alone gives a precipitate.

[1] Brit. Med. Journ., Oct. 17. [2] Med. Press and Circ., Dec. 16. [3] Brit. Med. Journ., Oct. 31. [4] Brit. Med. Journ., Nov. 7. [5] Med. Chronic., Aug. [6] Med. Press and Circ., Dec. 30. [7] Brit. Med. Journ., Oct. 10. [8] Brit. Med. Journ., Oct. 24. [9] Americ. Journ of Med. Sciences, Oct. [10] Brit. Med. Journ., Oct. 3. [11] Brit. Med. Journ., Dec. 12. [12] Med. Rec., Oct. 24. [13] New York Med. Journ., Sept. [14] Americ. Med. Surg. Bulletin, Oct. 3. [15] Les Nouv. Remédes, Sept. 8. [16] New York Med. Journ., Sept. 5.

